# 7-Fluoro-4-oxochromene-3-carbaldehyde

**DOI:** 10.1107/S1600536811007045

**Published:** 2011-03-02

**Authors:** Mohammad Asad, Chuan-Wei Oo, Hasnah Osman, Madhukar Hemamalini, Hoong-Kun Fun

**Affiliations:** a School of Chemical Sciences, Universiti Sains Malaysia, 11800 USM, Penang, Malaysia; b X-ray Crystallography Unit, School of Physics, Universiti Sains Malaysia, 11800 USM, Penang, Malaysia

## Abstract

In the title compound, C_10_H_5_FO_3_, the chromenone ring is essentially planar, with a maximum deviation of 0.039 (1) Å. The dihedral angle between the fluoro-subsituted benzene ring and the pyran ring is 1.92 (4)°. In the crystal, mol­ecules are connected *via* weak inter­molecular C—H⋯O hydrogen bonds, forming supra­molecular ribbons along the *b* axis. These ribbons are stacked down the *a* axis.

## Related literature

For the biological activity of chromones, see: Masami *et al.* (2007 ▶); Ellis *et al.* (1978 ▶); Raj *et al.* (2010 ▶); Nawrot-Modranka *et al.* (2006 ▶); Gomes *et al.* (2010 ▶). For the stability of the temperature controller used in the data collection, see: Cosier & Glazer (1986 ▶).
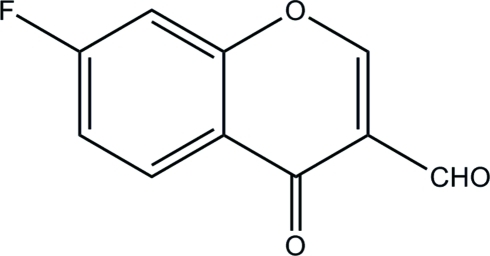


## Experimental

### 

#### Crystal data

C_10_H_5_FO_3_
*M* *_r_* = 192.14
Monoclinic, 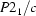

*a* = 3.7294 (1) Å
*b* = 6.2347 (2) Å
*c* = 34.6518 (11) Å
β = 90.740 (1)°
*V* = 805.65 (4) Å^3^
*Z* = 4
Mo *K*α radiation
μ = 0.13 mm^−1^
*T* = 100 K
0.52 × 0.20 × 0.08 mm

#### Data collection

Bruker APEXII DUO CCD area-detector diffractometer
Absorption correction: multi-scan (*SADABS*; Bruker, 2009 ▶) *T*_min_ = 0.935, *T*_max_ = 0.990
20369 measured reflections
2937 independent reflections
2622 reflections with *I* > 2σ(*I*)
*R*_int_ = 0.022

#### Refinement

*R*[*F* ^2^ > 2σ(*F* ^2^)] = 0.042
*wR*(*F* ^2^) = 0.125
*S* = 1.03
2937 reflections
127 parameters
H-atom parameters constrained
Δρ_max_ = 0.68 e Å^−3^
Δρ_min_ = −0.18 e Å^−3^

### 

Data collection: *APEX2* (Bruker, 2009 ▶); cell refinement: *SAINT* (Bruker, 2009 ▶); data reduction: *SAINT*; program(s) used to solve structure: *SHELXTL* (Sheldrick, 2008 ▶); program(s) used to refine structure: *SHELXTL*; molecular graphics: *SHELXTL*; software used to prepare material for publication: *SHELXTL* and *PLATON* (Spek, 2009 ▶).

## Supplementary Material

Crystal structure: contains datablocks global, I. DOI: 10.1107/S1600536811007045/rz2562sup1.cif

Structure factors: contains datablocks I. DOI: 10.1107/S1600536811007045/rz2562Isup2.hkl

Additional supplementary materials:  crystallographic information; 3D view; checkCIF report

## Figures and Tables

**Table 1:** Hydrogen-bond geometry (Å, °)

*D*—H⋯*A*	*D*—H	H⋯*A*	*D*⋯*A*	*D*—H⋯*A*
C1—H1*A*⋯O3^i^	0.93	2.39	3.2147 (11)	148
C3—H3*A*⋯O2^ii^	0.93	2.29	3.1419 (12)	152
C10—H10*A*⋯O3^iii^	0.93	2.58	3.3010 (14)	135

Symmetry codes: (i) 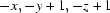; (ii) 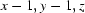; (iii) 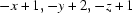.

**Fig. 1.**
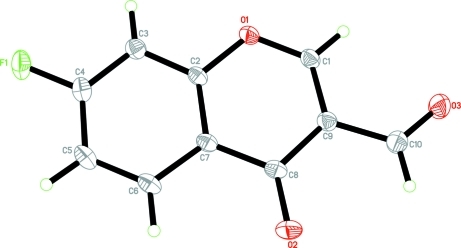
The asymmetric unit of the title compound, showing 50% probability displacement ellipsoids and the atom-numbering scheme.

**Fig. 2.**
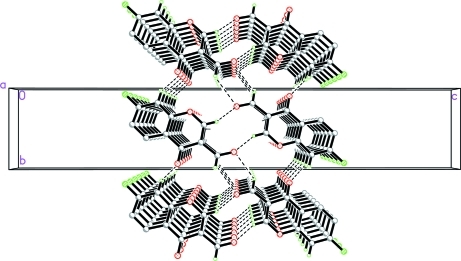
Crystal packing of the title compound viewed along the a axis, showing a hydrogen-bonded (dashed lines) network.

## supplementary crystallographic information

### Comment

A large number of chromones and their derivatives possess a broad range of biological activities such as anti-HIV (Masami *et al.*, 2007), antiallergic (Ellis *et al.*, 1978), anticancer (Raj *et al.*, 2010), antibacterial (Nawrot-Modranka *et al.*, 2006), antiviral and antioxidant (Gomes *et al.*, 2010) properties. We report here the structure of a newly synthesized chromone derivative, 7-fluoro-3-formylchromone. It was synthesized by own experimental process and structure elucidation was primarily  carried out by elemental analysis, ^1^H NMR and IR spectroscopic techniques.

The asymmetric unit of the title compound is shown in Fig. 1. The chromenone (O1/C1–C9) ring is essentially planar, with a maximum deviation of 0.039 (1) Å for atom C8. The dihedral angle between the fluoro-substituted benzene (C2–C7) ring and the pyran (O1/C1/C2/C7–C9) ring is 1.92 (4)°.

In the crystal structure (Fig. 2), adjacent molecules are connected via intermolecular C1—H1A···O3; C3—H3A···O2 and C10—H10A···O3 (Table 1) hydrogen bonds to form supramolecular ribbons along the *b* axis. These ribbons are stacked down the *a* axis.

### Experimental

To a well stirred solution of 4-fluoro-2-hydroxyacetophenone (6.5 mmol, 1.0 g) in DMF (4 ml), POCl_3_ (26.1 mmol, 2.4 ml) was added dropwise with stirring in ice bath. After 15 minutes, the ice bath was removed and the reaction mixture was continued to be stirred at room temperature for overnight. The resultant reaction mixture was then decomposed by crushed ice and the final product was collected by filtration, washed with ethanol-water and recrystallized from acetone to afford the title compound in 75% yield.

### Refinement

All the H atoms were positioned geometrically [ C–H = 0.93 Å ] and were refined using a riding model, with *U*_iso_(H) = 1.2 *U*_eq_(C). The highest peak in the final difference map was found at a distance of 0.68 Å from C3 and the deepest hole was 0.79 Å from C2.

### Crystal data

**Table:**

C_10_H_5_FO_3_	*F*(000) = 392
*M**_r_* = 192.14	*D*_x_ = 1.584 Mg m^−^^3^
Monoclinic, *P*2_1_/*c*	Mo *K*α radiation, λ = 0.71073 Å
Hall symbol: -P 2ybc	Cell parameters from 9357 reflections
*a* = 3.7294 (1) Å	θ = 3.3–32.6°
*b* = 6.2347 (2) Å	µ = 0.13 mm^−^^1^
*c* = 34.6518 (11) Å	*T* = 100 K
β = 90.740 (1)°	Plate, yellow
*V* = 805.65 (4) Å^3^	0.52 × 0.20 × 0.08 mm
*Z* = 4	

### Data collection

**Table:**

Bruker APEXII DUO CCD area-detector diffractometer	2937 independent reflections
Radiation source: fine-focus sealed tube	2622 reflections with *I* > 2σ(*I*)
graphite	*R*_int_ = 0.022
φ and ω scans	θ_max_ = 32.6°, θ_min_ = 1.2°
Absorption correction: multi-scan (*SADABS*; Bruker, 2009)	*h* = −5→5
*T*_min_ = 0.935, *T*_max_ = 0.990	*k* = −9→9
20369 measured reflections	*l* = −51→52

### Refinement

**Table:**

Refinement on *F*^2^	Primary atom site location: structure-invariant direct methods
Least-squares matrix: full	Secondary atom site location: difference Fourier map
*R*[*F*^2^ > 2σ(*F*^2^)] = 0.042	Hydrogen site location: inferred from neighbouring sites
*wR*(*F*^2^) = 0.125	H-atom parameters constrained
*S* = 1.03	*w* = 1/[σ^2^(*F*_o_^2^) + (0.0705*P*)^2^ + 0.3007*P*] where *P* = (*F*_o_^2^ + 2*F*_c_^2^)/3
2937 reflections	(Δ/σ)_max_ = 0.001
127 parameters	Δρ_max_ = 0.68 e Å^−^^3^
0 restraints	Δρ_min_ = −0.18 e Å^−^^3^

### Special details

**Table:**

Experimental. The crystal was placed in the cold stream of an Oxford Cryosystems Cobra open-flow nitrogen cryostat (Cosier & Glazer, 1986) operating at 100.0 (1) K.
Geometry. All s.u.'s (except the s.u. in the dihedral angle between two l.s. planes) are estimated using the full covariance matrix. The cell s.u.'s are taken into account individually in the estimation of s.u.'s in distances, angles and torsion angles; correlations between s.u.'s in cell parameters are only used when they are defined by crystal symmetry. An approximate (isotropic) treatment of cell s.u.'s is used for estimating s.u.'s involving l.s. planes.
Refinement. Refinement of F^2^ against ALL reflections. The weighted R-factor wR and goodness of fit S are based on F^2^, conventional R-factors R are based on F, with F set to zero for negative F^2^. The threshold expression of F^2^ > 2σ(F^2^) is used only for calculating R-factors(gt) etc. and is not relevant to the choice of reflections for refinement. R-factors based on F^2^ are statistically about twice as large as those based on F, and R- factors based on ALL data will be even larger.

### Fractional atomic coordinates and isotropic or equivalent isotropic displacement parameters (Å^2^)

**Table:**

	*x*	*y*	*z*	*U*_iso_*/*U*_eq_	
F1	0.11979 (18)	0.11518 (12)	0.279011 (18)	0.02856 (16)	
O1	0.12537 (18)	0.31079 (11)	0.409211 (19)	0.01826 (15)	
O2	0.6366 (2)	0.87750 (12)	0.38719 (2)	0.02381 (17)	
O3	0.2976 (2)	0.75507 (14)	0.49717 (2)	0.02861 (18)	
C1	0.1927 (2)	0.45457 (15)	0.43718 (2)	0.01768 (17)	
H1A	0.1255	0.4194	0.4621	0.021*	
C2	0.2137 (2)	0.36246 (14)	0.37194 (2)	0.01554 (16)	
C3	0.1252 (2)	0.20906 (15)	0.34431 (3)	0.01823 (17)	
H3A	0.0180	0.0796	0.3508	0.022*	
C4	0.2053 (2)	0.25964 (16)	0.30674 (3)	0.01986 (18)	
C5	0.3659 (2)	0.45082 (17)	0.29548 (3)	0.02122 (19)	
H5A	0.4134	0.4783	0.2697	0.025*	
C6	0.4525 (2)	0.59831 (16)	0.32376 (3)	0.01913 (18)	
H6A	0.5617	0.7268	0.3170	0.023*	
C7	0.3775 (2)	0.55659 (14)	0.36268 (2)	0.01570 (16)	
C8	0.4695 (2)	0.71039 (14)	0.39326 (3)	0.01698 (17)	
C9	0.3518 (2)	0.64737 (15)	0.43164 (3)	0.01704 (17)	
C10	0.4046 (3)	0.79260 (17)	0.46468 (3)	0.02236 (19)	
H10A	0.5266	0.9205	0.4606	0.027*	

### Atomic displacement parameters (Å^2^)

**Table:**

	*U*^11^	*U*^22^	*U*^33^	*U*^12^	*U*^13^	*U*^23^
F1	0.0312 (3)	0.0358 (4)	0.0187 (3)	−0.0032 (3)	0.0015 (2)	−0.0083 (2)
O1	0.0214 (3)	0.0184 (3)	0.0151 (3)	−0.0049 (2)	0.0026 (2)	0.0025 (2)
O2	0.0249 (3)	0.0205 (3)	0.0260 (3)	−0.0073 (3)	0.0031 (3)	0.0042 (3)
O3	0.0367 (4)	0.0295 (4)	0.0198 (3)	−0.0090 (3)	0.0056 (3)	−0.0039 (3)
C1	0.0181 (4)	0.0201 (4)	0.0149 (3)	−0.0024 (3)	0.0014 (3)	0.0023 (3)
C2	0.0140 (3)	0.0185 (4)	0.0141 (3)	−0.0006 (3)	0.0022 (3)	0.0030 (3)
C3	0.0164 (4)	0.0203 (4)	0.0180 (4)	−0.0014 (3)	0.0014 (3)	0.0008 (3)
C4	0.0169 (4)	0.0264 (4)	0.0162 (4)	0.0007 (3)	0.0000 (3)	−0.0022 (3)
C5	0.0182 (4)	0.0300 (5)	0.0155 (4)	0.0007 (3)	0.0024 (3)	0.0044 (3)
C6	0.0162 (4)	0.0233 (4)	0.0179 (4)	−0.0007 (3)	0.0025 (3)	0.0064 (3)
C7	0.0133 (3)	0.0178 (4)	0.0160 (3)	−0.0004 (3)	0.0012 (3)	0.0037 (3)
C8	0.0145 (3)	0.0178 (4)	0.0187 (4)	−0.0007 (3)	0.0016 (3)	0.0043 (3)
C9	0.0164 (3)	0.0185 (4)	0.0162 (3)	−0.0023 (3)	0.0006 (3)	0.0013 (3)
C10	0.0244 (4)	0.0230 (4)	0.0198 (4)	−0.0041 (3)	0.0010 (3)	−0.0013 (3)

### Geometric parameters (Å, °)

**Table:**

F1—C4	1.3521 (11)	C3—H3A	0.9300
O1—C1	1.3414 (11)	C4—C5	1.3921 (14)
O1—C2	1.3751 (10)	C5—C6	1.3791 (14)
O2—C8	1.2334 (11)	C5—H5A	0.9300
O3—C10	1.2218 (12)	C6—C7	1.4052 (12)
C1—C9	1.3551 (12)	C6—H6A	0.9300
C1—H1A	0.9300	C7—C8	1.4664 (13)
C2—C3	1.3902 (12)	C8—C9	1.4599 (12)
C2—C7	1.3950 (12)	C9—C10	1.4711 (13)
C3—C4	1.3759 (12)	C10—H10A	0.9300
			
C1—O1—C2	118.49 (7)	C5—C6—C7	120.72 (9)
O1—C1—C9	124.66 (8)	C5—C6—H6A	119.6
O1—C1—H1A	117.7	C7—C6—H6A	119.6
C9—C1—H1A	117.7	C2—C7—C6	118.33 (8)
O1—C2—C3	115.35 (8)	C2—C7—C8	120.02 (8)
O1—C2—C7	122.02 (8)	C6—C7—C8	121.65 (8)
C3—C2—C7	122.63 (8)	O2—C8—C9	122.77 (9)
C4—C3—C2	116.20 (9)	O2—C8—C7	122.90 (8)
C4—C3—H3A	121.9	C9—C8—C7	114.32 (8)
C2—C3—H3A	121.9	C1—C9—C8	120.34 (8)
F1—C4—C3	117.88 (9)	C1—C9—C10	119.36 (8)
F1—C4—C5	118.01 (8)	C8—C9—C10	120.30 (8)
C3—C4—C5	124.11 (9)	O3—C10—C9	123.87 (9)
C6—C5—C4	118.01 (8)	O3—C10—H10A	118.1
C6—C5—H5A	121.0	C9—C10—H10A	118.1
C4—C5—H5A	121.0		
			
C2—O1—C1—C9	−1.81 (13)	C5—C6—C7—C2	0.03 (13)
C1—O1—C2—C3	−177.90 (8)	C5—C6—C7—C8	179.56 (8)
C1—O1—C2—C7	1.50 (12)	C2—C7—C8—O2	174.82 (8)
O1—C2—C3—C4	178.80 (8)	C6—C7—C8—O2	−4.70 (14)
C7—C2—C3—C4	−0.59 (13)	C2—C7—C8—C9	−4.23 (12)
C2—C3—C4—F1	−179.36 (8)	C6—C7—C8—C9	176.24 (8)
C2—C3—C4—C5	0.09 (14)	O1—C1—C9—C8	−1.12 (14)
F1—C4—C5—C6	179.89 (8)	O1—C1—C9—C10	178.88 (9)
C3—C4—C5—C6	0.45 (15)	O2—C8—C9—C1	−175.04 (9)
C4—C5—C6—C7	−0.49 (14)	C7—C8—C9—C1	4.01 (12)
O1—C2—C7—C6	−178.81 (8)	O2—C8—C9—C10	4.95 (14)
C3—C2—C7—C6	0.54 (13)	C7—C8—C9—C10	−175.99 (8)
O1—C2—C7—C8	1.65 (13)	C1—C9—C10—O3	−3.67 (15)
C3—C2—C7—C8	−178.99 (8)	C8—C9—C10—O3	176.33 (10)

### Hydrogen-bond geometry (Å, °)

**Table:**

*D*—H···*A*	*D*—H	H···*A*	*D*···*A*	*D*—H···*A*
C1—H1A···O3^i^	0.93	2.39	3.2147 (11)	148
C3—H3A···O2^ii^	0.93	2.29	3.1419 (12)	152
C10—H10A···O3^iii^	0.93	2.58	3.3010 (14)	135

Symmetry codes: (i) −*x*, −*y*+1, −*z*+1; (ii) *x*−1, *y*−1, *z*; (iii) −*x*+1, −*y*+2, −*z*+1.
